# Effect of sulfur hexafluoride gas and post-annealing treatment for inductively coupled plasma etched barium titanate thin films

**DOI:** 10.1186/1556-276X-9-496

**Published:** 2014-09-15

**Authors:** Cong Wang, Yang Li, Zhao Yao, Hong-Ki Kim, Hyung-Jun Kim, Nam-Young Kim

**Affiliations:** 1 Department of Electronic Engineering, Kwangwoon University, 20 Gwangun-Ro, Nowon-gu, Seoul 139-701, Republic of Korea; 2 Department of Electronic Materials Engineering, Kwangwoon University, 20 Gwangun-Ro, Nowon-gu, Seoul 139-701, Republic of Korea

**Keywords:** Sulfur hexafluoride, Post-annealing treatment, Inductively coupled plasma etching, Barium titanate

## Abstract

Aerosol deposition- (AD) derived barium titanate (BTO) micropatterns are etched via SF_6_/O_2_/Ar plasmas using inductively coupled plasma (ICP) etching technology. The reaction mechanisms of the sulfur hexafluoride on BTO thin films and the effects of annealing treatment are verified through X-ray photoelectron spectroscopy (XPS) analysis, which confirms the accumulation of reaction products on the etched surface due to the low volatility of the reaction products, such as Ba and Ti fluorides, and these residues could be completely removed by the post-annealing treatment. The exact peak positions and chemicals shifts of Ba *3d*, Ti *2p*, O *1 s*, and F *1 s* are deduced by fitting the XPS narrow-scan spectra on as-deposited, etched, and post-annealed BTO surfaces. Compared to the as-deposited BTOs, the etched Ba *3d*_*5*/*2*_, Ba *3d*_*3*/*2*_, Ti *2p*_*3*/*2*_, Ti *2p*_*1*/*2*_, and O *1 s* peaks shift towards higher binding energy regions by amounts of 0.55, 0.45, 0.4, 0.35, and 0.85 eV, respectively. A comparison of the as-deposited film with the post-annealed film after etching revealed that there are no significant differences in the fitted XPS narrow-scan spectra except for the slight chemical shift in the O *1 s peak* due to the oxygen vacancy compensation in O_2_-excessive atmosphere. It is inferred that the electrical properties of the etched BTO film can be restored by post-annealing treatment after the etching process. Moreover, the relative permittivity and loss tangent of the post-annealed BTO thin films are remarkably improved by 232% and 2,695%, respectively.

## Background

Today, ferroelectric thin films have been identified as a promising candidate material for capacitors in the next generation ultra-large-scale integrated dynamic random access memories, infrared sensors, electro-optic, RF, and microwave devices [1-3]. Among the numerous ferroelectrics, barium titanate (BTO) thin films are known as one of the leading candidates for use in applications due to their high dielectric constant, low leakage current, lack of fatigue, and low crystallization temperature [4-6]. Currently, a new, green and environmentally friendly approach called aerosol deposition (AD) has attracted great interest to fabricate BTO, which is a low-temperature and low-cost method featuring room-temperature processing with high deposition rate and high density [7-9]. To realize highly integrated microelectronic silicon-based devices involving BTO films, dry etching processes should be developed [10,11]. However, there is currently no feasible technology known for the etching of BTO films. In addition, the data concerning the etching properties of such films exhibit significant deviations from the results obtained from other studies conducted under similar conditions [12]. These obstacles have led to a misunderstanding of the basic effects of the BTO etching process and have hindered the optimization of this process. Therefore, the majority of the work conducted to understand the etching mechanisms has focused on elucidating the role of both physical and chemical etching effects. However, even after the SF_6_/O_2_/Ar-based over-etching process, a large amount of sticky black by-products (BaF_2_ and TiF_4_) is still observed on the desired etching area; moreover, BTO thin films exhibit relatively inferior electric properties than those in their bulk ceramic and single crystal counterparts [13]. The above-mentioned factors are influenced by the post-annealing process, which is essential for the cleanup of the by-products during the etching process and the densification of the BTO thin films. High annealing temperature is inevitable because the low-volatility compound BaF_2_ requires more energy to break the Ba-F bonds [14]. Additionally, the annealing duration is indispensable for obtaining good quality crystalline BTO films [15,16].

In this work, the etching characteristics and mechanisms of BTO films in SF_6_/O_2_/Ar plasma and the effect of post-annealing treatment are investigated in detail on the nanoscale structural, physical, chemical, and microwave dielectric properties. The chemical compositions and the binding states on the surface of the BTO films are analyzed by X-ray photoelectron spectroscopy (XPS). The exact peak positions and the chemical changes in the elements of interest are deduced by fitting the XPS narrow-scan spectra of Ba *3d*, Ti *2p*, O *1 s*, and F *1 s* for each BTO sample. The experimental results indicate that there are no significant differences between the as-deposited BTO film and the post-annealed film after etching with respect to the fitted narrow-scan spectra. Through post-annealing in an O_2_-rich environment for 2 h, an improvement in the dielectric properties of BTO thin films is achieved. Based on these results, annealing treatment of the BTO thin film after the etching process is carried out for the room-temperature-deposited AD-based BTO thin films. A satisfactory etching scheme along with appropriate electrical properties are required for high-K dielectric materials to be used in the production of metal-insulator-metal capacitors in integrated passive devices (IPDs) [17,18] and high-K gate dielectric in high electron mobility transistor (HEMT) applications [19,20].

## Methods

The BTO thin films are deposited onto Pt/Ti/SiO_2_/silicon substrates via an AD process using a commercial 300 nm BTO powder (SBT-03B, Samsung Fine Chemicals Co., Ltd., Ulsan, South Korea) as the starting material. The particles are aerosolized in an aerosol chamber and transported into a deposition chamber using 5 L/min N_2_ gas. The transported BTO powders are continuously ejected through the nozzle and deposited onto the silicon substrate. The orifice size of the nozzle, the deposition area, the distance between the nozzle and the substrate, the working pressure, and the deposition time are 10 × 0.4 mm^2^ (10 mm wide, 0.4 mm slit width), 10 × 10 mm^2^, 5 mm, 3.4 Torr, and 10 min, respectively. The final thickness of each BTO film is approximately 1 μm. A negative photoresist, specifically 3.5-μm thick DNR-L300-40 (Dongjin Semichem Co., Ltd., Seoul, South Korea), is spun at 5,000 rpm for 40 s in the track before being baked for 90 s at 90°C. Afterwards, a photolithographic process is performed with an exposure energy of 120 mJ/cm^2^, and then a post-exposure bake is performed for 90 s at 100°C. The photoresist is then developed in an AZ300MIF developer (AZ Electronic Materials USA Corp., NJ, USA) for 60 s. Next, a 10/1,490-nm Ti/Cr metal shadow mask is fabricated by e-beam evaporation and used during the BTO etching. After the metallization process, the photoresist is stripped using acetone. The BTO films are etched in an inductively coupled plasma (ICP) etching system (STS Multiplex ICP ASE Etcher, Surface Technology Systems Ltd., Wales, United Kingdom). The flow rates of the SF_6_, O_2_, and Ar gases into the operation chamber are controlled by mass flow controllers. The BTO films are etched under the flow rates of SF_6_/O_2_/Ar of 75/5/10 sccm. Finally, the Ti/Cr shadow mask is stripped using hydrofluoric acid and a Cr etchant; the etched BTO films are post-annealed at 750°C with the rate of up and down temperature of 3°C/min for 2 h under O_2_ atmosphere via mini furnace annealing (SJVF-2100, Sungjin-Semitech Co., Siheung, South Korea) to completely remove all of the fluoride formed during the etching process.

**Figure 1:**
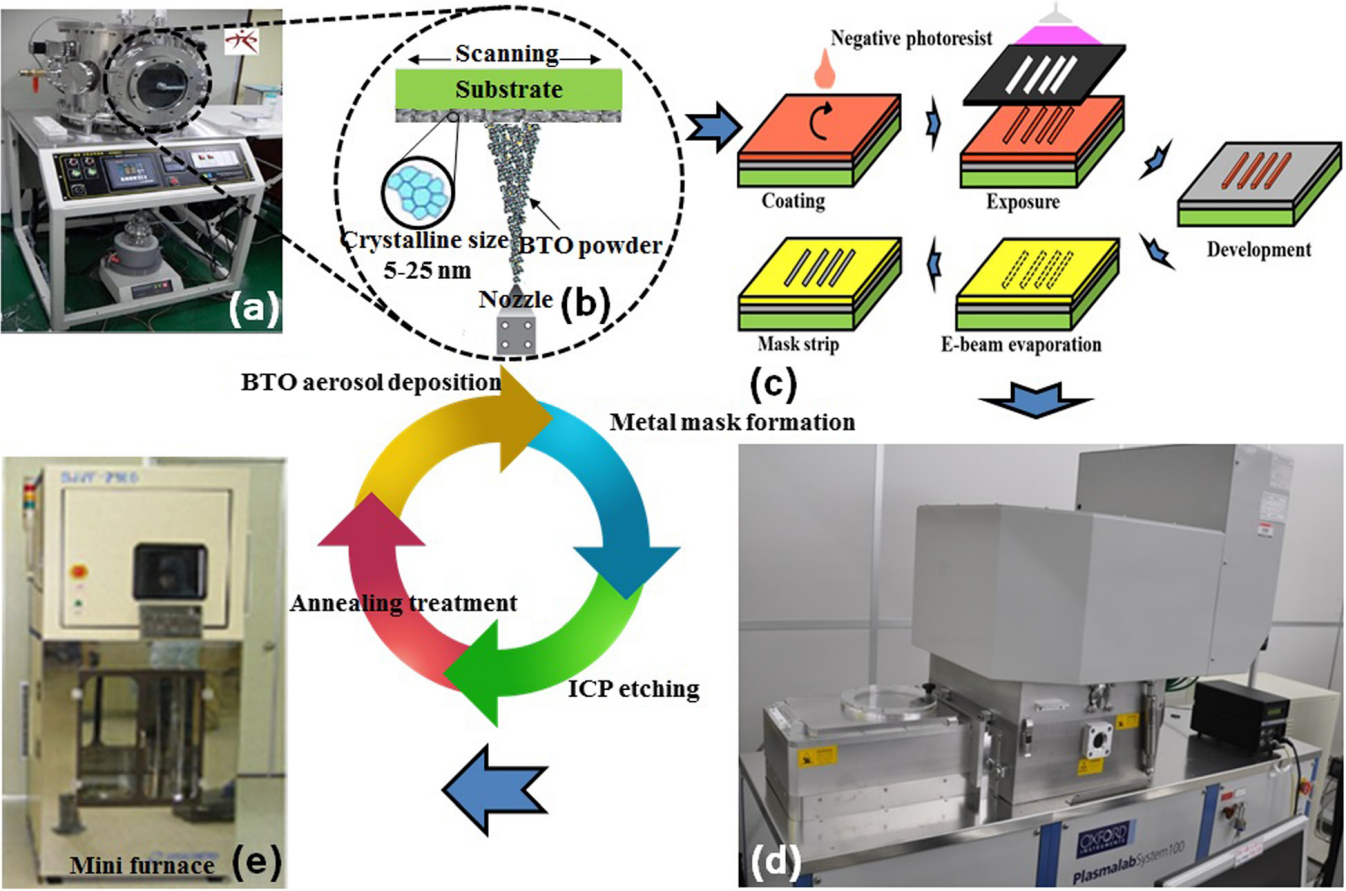
**The schematic diagram of this study. (a)** The aerosol deposition instrument in our laboratory, **(b)** schematic of the room-temperature growth of the BTO thin film on a substrate, **(c)** the fabrication flow of the preparation of Ti/Cr shadow metal mask, **(d)** the ICP etching equipment in KANC, and **(e)** the mini furnace machine (courtesy of Inter-university Semiconductor Research Center of Seoul National University).

The surface morphology of the same BTO film under the as-deposited, etched, and post-annealed after-etched circumstances is examined using atomic force microscopy (AFM; XE-100, PSIA Co., Suwon, South Korea). The chemical reactions and surface states of the BTO films are analyzed using XPS; an Al-K_α_ radiation source provides non-monochromatic X-rays at 1,486.6 eV. Survey spectra are obtained at a base pressure of 1.1 × 10^-7^ Pa, and a binding energy scan range of 1,000 to 0 eV is sufficient to identify all of the detectable elements. Narrow-scan spectra of all regions of interest are recorded with a pass energy of 23.5 eV to quantify the surface composition and identify the chemical binding state of the films. The C *1 s* peak at 285 eV is assigned to carbon due to hydrocarbon contamination and is used as the criterion to correct the energy of the spectra. PHI MultiPakTM software (Physical Electronics Inc., MN, USA) is used to fit the narrow-scan spectra of Ba *3d*, Ti *2p*, O *1 s*, and F *1 s* for as-deposited, etched, and post-annealed BTO films under Shirley-type background subtraction [21]. The crystal structure of the as-deposited and post-annealed BTO thin films are studied by X-ray diffraction (XRD) patterns, and then the crystallite sizes are calculated via Scherrer's formula. To measure the dielectric properties, upper electrodes of gold are coated by sputtering onto the BTO thin films over an area of 1.5 mm in diameter and lower electrodes are constructed by Pt/Ti-sputtered SiO_2_/Si substrate. Finally, the dielectric properties of the samples are measured from 10 Hz to 10 kHz by an impedance analyzer (E4991A, Agilent, CA, USA). The experimental work-flow of this study is shown in Figure 1.

## Results and discussion

### Etching mechanism

The performance of SF_6_/O_2_/Ar plasma must be understood because the etching process is affected by changes in the chemically reactive radicals and ions on the etching surface of the BTO films [22,23]. The major chemical reactions potentially involved in the BTO etching process are as follows:

(1) $$SF_{6}+\mathrm{energy}\;\mathrm{from}\;\mathrm{an}\;\mathrm{energised}\;\mathrm{particle}\to SF_{5}^{+}+\;F^{*}+\;2e$$

(2) $$SF_{5}^{+}\;+\;O\;+e\to \mathrm{SO}F_{4\;}\;↑+\;F^{*}$$

(3) $$\mathrm{Ar}\;+e\to Ar^{+}+\;2e$$

(4) $$\mathrm{BaTi}O_{3}\;+\;\left(2\;+\;x+\;3y\right)F^{*}\to^{↑}\mathrm{Ba}F_{2}+↑\mathrm{Ti}F_{x}\;+3OF_{y}$$

where *x* and *y* are unknown variables (Ti has oxidation states = +1, +2, +3, and +4, and the known chemical compounds of F and O are OF_2_, O_2_F_2_, and O_3_F_2_), *e* is an electron, and F^*^ is a fluoride atom, which is highly reactive. The long arrow indicates a highly volatile substance, while the short arrow denotes a somewhat volatile material. We assume that the mechanism of BTO etching involves Ar^+^-based physical sputtering etching and an F^*^-assisted chemical reaction; the etching effect is enhanced because O_2_ acts as a catalyst, and the low-volatility compounds, including BaF_2_ and TiF_*x*_, adhere to the etched surface due to a charging effect.

### Surface morphology

**Figure 2:**
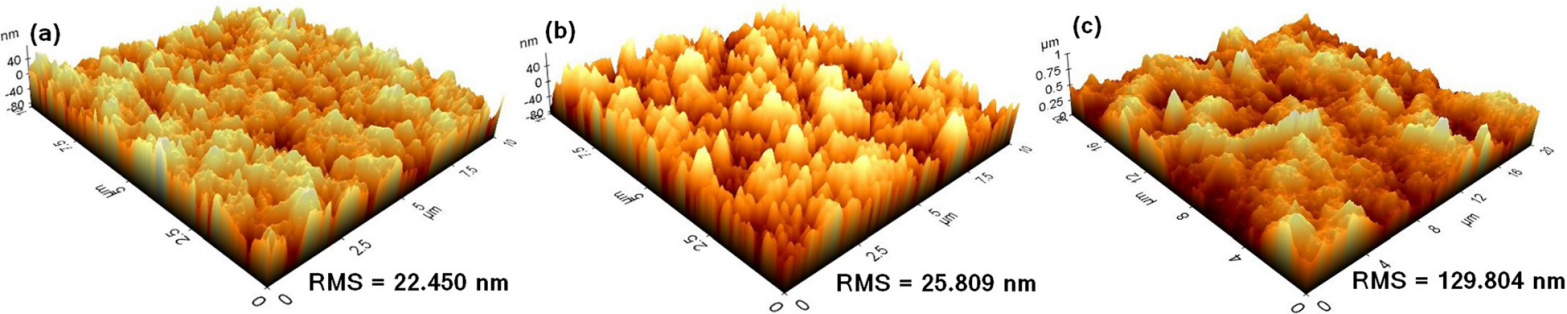
**Surface morphologies of the same BTO film measured using AFM. (a)** As-deposited, **(b)** etched in the SF_6_/O_2_/Ar (75/5/10 sccm) plasma, and **(c)** post-annealed at 750°C for 2 h under O_2_ ambient after the etching process.

Figure 2 shows AFM images of the same BTO film when under the as-deposited, etched, and post-annealed circumstances. The AFM images clearly indicate the roughness variations of the BTO films before and after the annealing treatment. The AFM images reveal that crystallite size grows, and larger grains start to form in the BTO thin film after the annealing process (Figure 2c) compared to those formed in other films. Note that surface structure of the as-deposited film has valleys with a moderate depth and that the valleys are densely distributed, as shown in Figure 2a, due to the deposition mechanism of the AD method. Additionally, the etched BTO film has a root-mean-square (RMS) of 25.809 nm in comparison with a RMS of approximately 22.450 nm for the same as-deposited film. Obviously, the compactness of the BTO film is deteriorated after being etched in SF_6_/O_2_/Ar plasma, as shown in Figure 2b. The increase in the surface roughness occurs primarily because low-volatility reaction products, such as (Ba, Ti) fluorides and fluorocarbon, are generated and scattered in the etching region and form a microscopic mask. Generally, when more fluorine ions participate in the reaction, more products are created and etching surface becomes rougher. The post-annealed after-etched surface (RMS = 129.804 nm) becomes terribly rugged in comparison with the etched surface prior to annealing, and lots of reaction residues remain on the surface, as shown in Figure 2c. This result indicates that the sintering phenomenon during annealing process, in which individual nanostructures merge together to form large structures, becomes important when materials are heated to elevated temperatures [24]. It could be noted that the surface morphologies deteriorate rapidly after post-annealing treatment; however, we have already developed an available solution by using a two-slurry-based chemical mechanical polishing method to overcome it with a possible RMS of less than 2 nm for the post-annealed BTO thin films.

### Chemical composition

**Figure 3:**
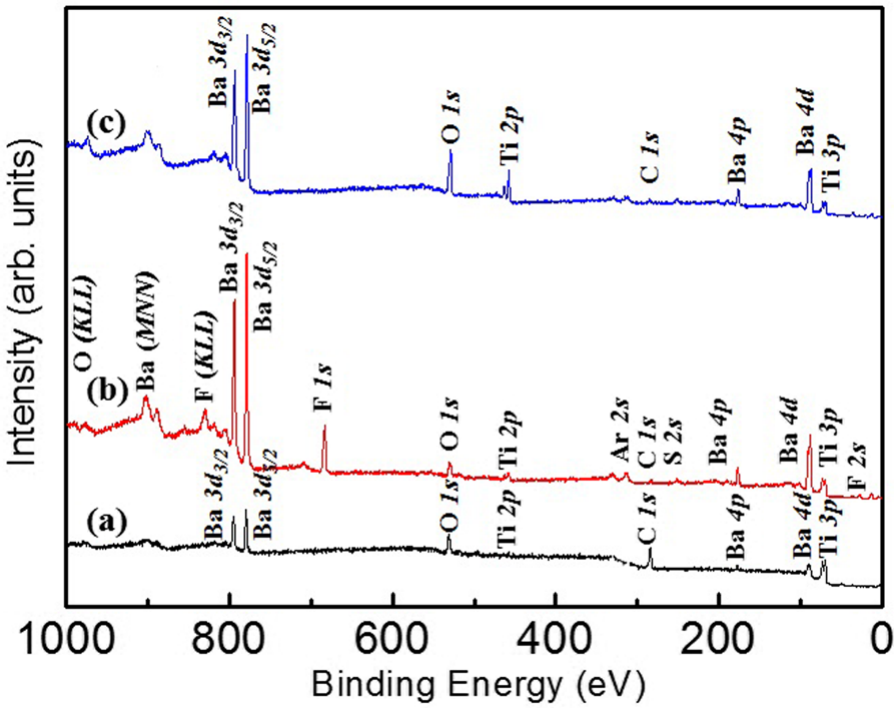
**XPS survey spectra of the BTO films. (a)** As-deposited BTO, **(b)** BTO etched with the SF_6_/O_2_/Ar plasma, and **(c)** etched BTO after the post-annealing process.

The XPS survey spectra obtained for the as-deposited, etched, and post-annealed BTO films are shown in Figure 3. In Figure 3a, the as-deposited BTO films are shown to contain the elements Ba, Ti, O, and C near their surface. The peak at 285.0 eV is assigned to C *1 s* due to hydrocarbon contamination. This peak is used as a criterion for rectifying the energy axis of the spectra [25]. There are XPS photoelectron lines of the elements Ba, Ti, C, O, F, S, and Ar near the SF_6_/O_2_/Ar-etched BTO surface: Ba *4d* (89.4 eV), Ba *4p* (185.7 eV), Ti *3p* (73.2 eV), C *1 s* (285.0 eV), S *2 s* (247.5 eV), F *2 s* (29.4 eV), and Ar *2 s* (313.5 eV), and the valence-type Auger lines for F (*KLL*) (838.2 eV), Ba (*MNN*) (902.7 eV), and O (*KLL*) (990.3 eV) can be identified in Figure 3b. Clearly, there is no photoelectron line of the element F in the as-deposited BTO specimen. After etching in an SF_6_/O_2_/Ar environment, the F *1 s* XPS spectrum exhibits a wide peak in the region of 682 to 686 eV, with a maximum corresponding to a binding energy of 683.1 eV. It is possible for Ar^+^ ions to be entrapped in the presence of cation vacancies or donor impurities present on the etched surface. The presence of Ba, Ti, and O signals is shown in the etched surface, while there is an absence of signal for both F and Ar for the etched BTO film after the annealing treatment, as shown in Figure 3c. That is, the elements of F and Ar are completely removed from the etched BTO surface by performing the post-annealing step in O_2_ ambient after etching.

### Fitted XPS narrow-scan spectra

**Figure 4:**
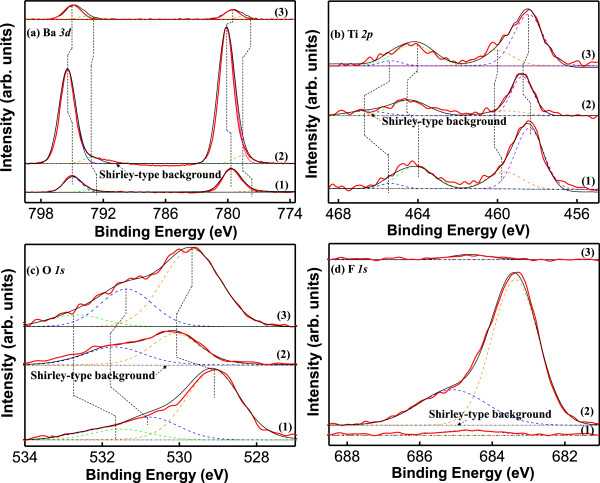
**The fitted narrow-scan spectra. (a)** Ba *3d*, **(b)** Ti *2p*, **(c)** O *1 s*, and **(d)** F *1 s* peaks for each BTO film: (1) as-deposited BTO, (2) BTO etched with SF_6_/O_2_/Ar (75/5/10) plasma, and (3) etched BTO after post-annealing treatment. Red solid lines represent the experimental results, black solid lines represent the fitted results after subtracting the Shirley-type background, and dashed peaks in blue, violet, green, and orange are sub-peaks fitted using PHI MultiPakTM software. The peak positions of the Ba *3d*, Ti *2p*, O *1 s,* and F *1 s* are the average peak positions for the corresponding sub-peaks. The broken lines indicate the approximate chemical shifts.

The fitted narrow-scan spectra of the Ba *3d*, Ti *2p*, O *1 s*, and F *1 s* peaks for each BTO thin film are shown in Figure 4. Figure 4a (1) shows that the as-deposited Ba *3d* doublet consists of two peaks at 779.05 and 794.1 eV, with a spin-orbit splitting energy (Δ) of 15.05 eV, which are mainly identified as a signal from Ba-O bonds, with the sub-peaks of Ba *3d*_*5*/*2*_ and Ba *3d*_*3*/*2*_ related to BaCO_3_ or a relaxed Ba phase due to O vacancies which are the cation defects [25,26]. Figure 4a (2) demonstrates that, compared to the as-deposited Ba *3d* doublet, after the etching process under the SF_6_/O_2_/Ar plasma treatment, the Ba *3d*_*5*/*2*_ (779.8 eV) and Ba *3d*_*3*/*2*_ (794.75 eV) peaks of the BTO film shift towards higher binding energy regions by 0.75 and 0.65 eV, respectively, because the bonding energies of the Ba-F bonds are higher than those of the Ba-O bonds, which is due to the higher electronegativity of F relative to that of O [27]. These chemical shifts confirm that some Ba-O bonds are broken and a small number of Ba-F bonds are generated. Compared to Figure 4a (2), the Ba *3d*_*5*/*2*_ and Ba *3d*_*3*/*2*_ peaks of the post-annealed BTO exhibit only slight chemical shifts towards lower binding energy regions, which indicate that Ba-F bonds are destroyed and Ba-O bands are regenerated under O_2_-rich ambience after the annealing process.

The fitted Ti *2p* narrow-scan spectra of the as-deposited BTO film are demonstrated in Figure 4b (1), which is composed of two wide peaks of Ti *2p*_*3*/*2*_ and Ti *2p*_*1*/*2*_ attributed to Ti-O bonds with binding energies of 458.4 and 464.1 eV, respectively. The Δ value of the Ti *2p* doublet is equal to 5.7 eV, which is comparable to the theoretical value (ΔTi *2p*) of Ti for Ti oxide. After etching, the intensity of the Ti-O peaks decreases due to the volatility of TiF_x_, which is partly removed from the surface during the thermal desorption process. The suppressed peak shifts to higher binding energy regions by 0.4 and 0.35 eV for of Ti *2p*_*3*/*2*_ and Ti *2p*_*1*/*2*_, respectively, as shown in Figure 4b (2). This result can be explained by a bond shift compensation scheme between TiF_x_ and the etched BTO, in which the Ti^4+^ cations are partially reduced to create Ti^x+^ (x = 1,2,3) cations in the presence of adequate oxygen vacancies [24]. The peak intensities of Ti *2p*_*3*/*2*_ (458.5 eV) and Ti *2p*_*1*/*2*_ (464.15 eV) are strengthened when compared to the etched counterparts, as shown in Figure 4b (3), and return identically to their original binding energies found in the case before the etching process.

A broad O *1 s* peak (529.15 eV) of the as-deposited BTO film contains three sub-peaks located at 529.1, 530.8, and 531.5 eV, as revealed in Figure 4c (1). Because the BTO film consists of two components (BaO and TiO_2_) in a BTO solid solution and C-O bonds due to surface contamination, the sub-peaks are mainly ascribed to Ba-(O *1 s*) (780 eV), Ti-(O *1 s*)_2_ (529 eV), and C-(O *1 s*) (532.3 eV) bonds [25,28,29]. The significant shoulder is attributed to O vacancies and surface species, such as H_2_O and CO_2_, adsorbed from the air during the AD process. In Figure 4c (2), the C-O bands disappear after the etching process. This result indicates that the formation of the C-O bands is limited to a near-surface area of the film. The other O *1 s* spectra display a chemical shift to a higher binding energy area, which can be attributed to the disconnection of some of the Ba-(O *1 s*) and Ti-(O *1 s*)_2_ bonds due to the physical sputtering of Ar^+^ ions and the chemical reaction with F^*^ during the etching process. In Figure 4c (3), there is a small chemical shift in comparison with the as-deposited O *1 s* because there is an oxygen vacancy compensation on the etched surface during the post-annealing process in O_2_-excessive environment; three sub-peaks, as in the as-deposited BTO thin film, were observed.

The fitted F *1 s* narrow-scan spectra of the as-deposited BTO film are exhibited in Figure 4d (1). As expected, the spectra do not show signals from a fluorine-containing compound. Adding SF_6_/O_2_/Ar as the etching reaction gas is accompanied by the appearance of a F *1 s* peak with a binding energy of 683.1 eV, as demonstrated in Figure 4d (2). The sub-peaks are situated at 682.95 and 685.1 eV, which are assigned to the product of the etching reaction of Ba-(F *1 s*)_2_ (684.5 eV) and a residue of Ti-(F *1 s*)_4_ (684.9 eV), respectively [28,30]. In the view of the narrow-scan F *1 s* spectrum of the post-annealed BTO thin film in Figure 4d (3), there are no detected signals of fluorides from the etched BTO surface because all of the fluorides are vaporized after the post-annealing treatment.

### Dielectric property

**Figure 5:**
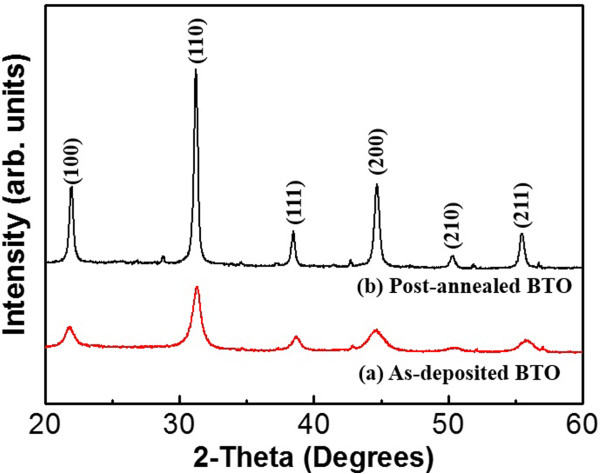
**XRD data of BTO films. (a)** as-deposited BTO and **(b)** post-annealed BTO.

**Figure 6:**
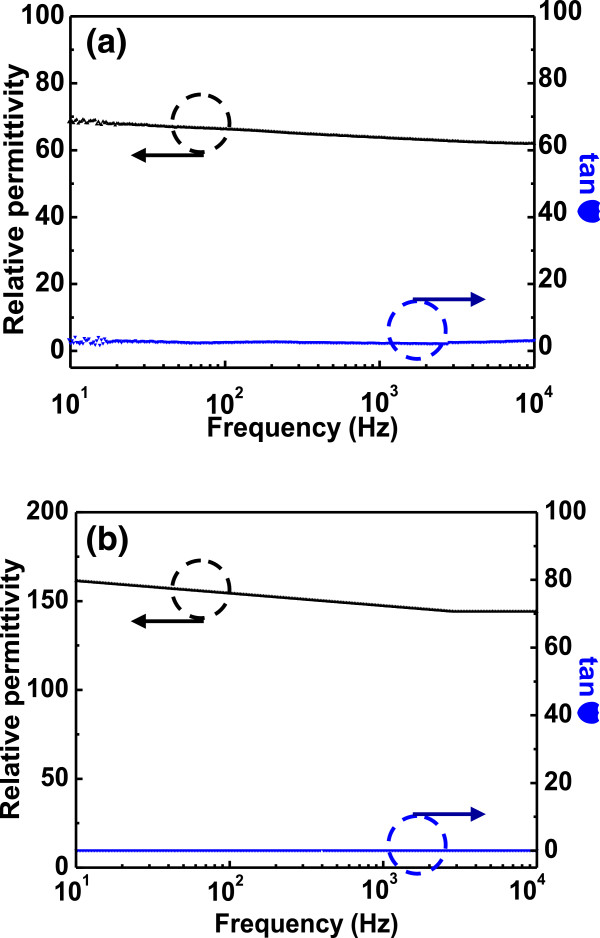
**Dielectric properties of BTO films. (a)** As-deposited BTO and **(b)** post-annealed BTO.

The XRD profiles confirm that both the as-deposited and the post-annealed BTO thin films have a cubic phase with a single perovskite structure, as shown in Figure 5. The full-width at half-maximum (FWHM) of the X-ray rocking curve reveals high values for as-deposited BTO (0.734) when compared to post-annealed BTO (0.295), and hence, the crystallite sizes of the as-deposited and post-annealed BTO are 11.3 and 29.9 nm, respectively, which are obtained using Scherrer's equation. Furthermore, there is no shift in the peak position, as is observed in films that undergo high-temperature post-annealing treatment. Thus, the XRD results clearly indicate that the annealing treatment promotes crystallization and retains nanocrystallinity. The relative permittivity and loss tangent of the as-deposited and post-annealed BTO thin films, measured as function of frequency at room temperature, are shown in Figure 6. The tan δ of as-deposited BTO thin film is confirmed to be higher (3.18) than that of post-annealed BTO thin films (0.118). As a result, the impurities related to the dielectric loss can be removed through the post-annealing process with grain growth [31]. The dielectric constant of the BTO thin films prepared under the post-annealing (144.08) exceeds those as-deposited ones (61.95). This result may be attributed to the preferred orientation of (100) in the thin film prepared under post-annealing process [32]. The dielectric constant is slightly decreased as the frequency increases, which is mostly due to the grains and grain boundaries by generating electrons and doubly ionized oxygen vacancies during the post-annealing process. From the above results, the importance of enhanced grain boundaries by doping acceptors is confirmed. Furthermore, we could verify the possibility to propose optimized dopant materials and their ration by investigation of the intrinsic dielectric properties of the AD-based BaTiO_3_ films without voids and ununiform grain sizes. Consequently, the post-annealing treatment is critically important for the room-temperature fabrication of AD-based BTO thin films with a low dielectric loss and a high dielectric constant.

## Conclusions

AD-derived BTO thin films are etched by sulfur hexafluoride ICP technology. The AFM images show that the roughness of the etched surface becomes rugged in comparison with the as-deposited surface because of the generation of the fluoride. After the post-annealing treatment, cross grain boundary diffusion becomes significant and the crystallite size grows, which caused a poor surface smoothness when compared with that of the other samples. The chemical compositions and bonding states for each BTO sample were studied by XPS. The low-volatility compound BaF_2_ was observed after the etching process and is due to a chemical reaction with F* after the destruction of O bonds via ion bombardment. Thus, the Ti-O bonds are destroyed by chemical reaction and the products of TiF_4_ are formed, and all of these residues could be removed by post-annealing process. The XRD studies indicated that the BTO thin films are well crystallized and have a preferred orientation of (100) after post-annealing process, which help to improve the dielectric properties. The post-annealed BTO thin films exhibit higher dielectric constant and lower dielectric loss compared to the as-deposited ones, which is important for practical device applications. This result leads to the conclusion that the post-annealing process is a cost-effective and appropriate method for both effectively removing etching by-products and obtaining nanocrystalline films, which plays an important role on the disposal of etching residue and dielectric characteristics of BTO thin films.

## Competing interests

The authors declare that they have no competing interests.

## Authors’ contributions

CW conceived of the study, managed the entire study, and drafted the manuscript. YL, ZY, H-KK, and H-JK performed the fabrication and the measurements. As the corresponding author, N-YK provided the overall research conception, guided the research, and revised the manuscript. All authors read and approved the final manuscript.
